# Proposal to Screen for Zinc and Selenium in Patients with IgA Deficiency

**DOI:** 10.3390/nu15092145

**Published:** 2023-04-29

**Authors:** Soraya Regina Abu Jamra, Camila Gomes Komatsu, Fernando Barbosa, Persio Roxo-Junior, Anderson Marliere Navarro

**Affiliations:** 1 Department of Pediatrics, Ribeirão Preto Medical School—University of São Paulo—FMRP/USP, Sao Paulo 05508-090, Brazil; sajamra@hcrp.usp.br; 2 Department of Food and Nutrition, Faculty of Pharmaceutical Sciences, São Paulo State University UNESP, Araraquara 14800-060, Brazil; camila_komatsu@yahoo.com.br; 3 Laboratory of Toxicology and Metal Essentiality, Faculty of Pharmaceutical Sciences of Ribeirão Preto, University of São Paulo—USP, Sao Paulo 05508-090, Brazil; fbarbosa@fcfrp.usp.br; 4 Department of Health Sciences, Division of Nutrition and Metabolism, Ribeirão Preto Medical School—University of São Paulo—FMRP/USP, Sao Paulo 05508-090, Brazil; navarro@fmrp.usp.br

**Keywords:** IgA deficiency, selenium deficiency, zinc deficiency, recommended dietary allowances, immune system

## Abstract

The increase in life expectancy can be a consequence of the world’s socioeconomic, sanitary and nutritional conditions. Some studies have demonstrated that individuals with a satisfactory diet variety score present a lower risk of malnutrition and better health status. Zinc and selenium are important micronutrients that play a role in many biochemical and physiological processes of the immune system. Deficient individuals can present both innate and adaptive immunity abnormalities and increased susceptibility to infections. Primary immunodeficiency diseases, also known as inborn errors of immunity, are genetic disorders classically characterized by an increased susceptibility to infection and/or dysregulation of a specific immunologic pathway. IgA deficiency (IgAD) is the most common primary antibody deficiency. This disease is defined as serum IgA levels lower than 7 mg/dL and normal IgG and IgM levels in individuals older than four years. Although many patients are asymptomatic, selected patients suffer from different clinical complications, such as pulmonary infections, allergies, autoimmune diseases, gastrointestinal disorders and malignancy. Knowing the nutritional status as well as the risk of zinc and selenium deficiency could be helpful for the management of IgAD patients. Objectives: to investigate the anthropometric, biochemical, and nutritional profiles and the status of zinc and selenium in patients with IgAD. Methods: in this descriptive study, we screened 16 IgAD patients for anthropometric and dietary data, biochemical evaluation and determination of plasma and erythrocyte levels of zinc and selenium. Results: dietary intake of zinc and selenium was adequate in 75% and 86% of the patients, respectively. These results were consistent with the plasma levels (adequate levels of zinc in all patients and selenium in 50% of children, 25% of adolescents and 100% of adults). However, erythrocyte levels were low for both micronutrients (deficiency for both in 100% of children, 75% of adolescents and 25% of adults). Conclusion: our results highlight the elevated prevalence of erythrocyte zinc and selenium deficiency in patients with IgAD, and the need for investigation of these micronutrients in their follow-up.

## 1. Introduction

Growth varies during life and it is recognized as a good indicator of a child’s health [1]. Robust evidence has shown the important role of diet in the maintenance of human health, as approximately one in five deaths around the world due to chronic cardiovascular and neoplastic diseases can be attributed to an unhealthy diet [2]. Therefore, to empower people to choose healthy foods and to create healthy environments has become one of the main global objectives of health policies [3].

Optimal nutrition plays a fundamental role in the adequate performance of the immune system. An adequate supply of nutrients is essential for immune cells proliferation and biosynthesis of several immune factors [4]. Micronutrients participate and support every stage of the response against pathogens, and have specific antibacterial and anti-viral functions. Micronutrients also act as substrates for the intestinal microbiota, regulate the immune cell metabolism, protect from oxidative and inflammatory stress and contribute to the production of proteins (antibodies, cytokines, receptors) and new cells [5]. It is well known that proper nutrition can help support optimal immune function, reducing the frequency and severity of infectious diseases. Inadequate dietary intake of some nutrients and malnutrition of a child or an adult can cause chronic inflammation due to dysregulation of the immune response and recurrent infections as a consequence of secondary immunodeficiency [5,6]. Childs et al. [7] have shown that malnourished individuals can present impaired phagocytosis and decreased cytokine production. On the other hand, chronic infection can lead to nutrient deficiencies through reduced appetite, greater consumption and reduced nutrient absorption, resulting in a vicious cycle of malnutrition and infection [8]. Moreover, according to the World Health Organization, there is a synergistic relationship between malnutrition and infection, in which the immune response exacerbates a poor nutritional state and causes an increase in the demand for micronutrients [9]. As a similar form, the hidden hunger, defined as an inadequate micronutrient intake, in contrast to an adequate or even excessive energy consumption, can also compromise the immune response [10]. Thus, an adequate dietary intake rich in some nutrients like protein, copper, iron, selenium and zinc has a significant role in immune health improving the quality of life [4].

Zinc (Zn) is one of the most abundant micronutrients in the human body. As a component of several enzymes and transcription factors, Zn is involved in many biochemical and physiological processes at the molecular, cellular and systemic levels. Zn participates in cell membrane repair, cell proliferation, inflammation, DNA damage response and antioxidant defenses [11]. Deficiency of Zn is related to the pathophysiology of many human conditions, ranging from cancer to neurological disorders, impairment in cognitive function, aging, diabetes, growth retardation and infection [12,13]. Zn also has antioxidant functions and plays a significant role in the maintenance of immune homeostasis. In the innate immune system, Zn is important for phagocytosis; it affects the production of cytokines, the maturation of dendritic cells and the activity of the complement system [14]. Regarding adaptive immunity, Zn influences the formation and function of T cells, as well as the maturation of B cells, and consequently antibody production. Zn is also crucial for the balance between the different T-cell subsets, since its deficiency decreases the production of Th1 cytokines (IFN-γ, IL-2 and TNF-α) that are essential for the adequate response against pathogens [14,15].

Selenium (Se) is present in 25 human selenoproteins involved in a variety of essential biological functions, ranging from the regulation of reactive oxygen species (ROS) to the biosynthesis of hormones [16]. Selenoprotein-mediated biochemical mechanisms play an important role in the clinical outcome of diseases that include cancer, diabetes, viral infections (including SARS-CoV-2 and HIV) and neurological disorders. Se can affect both the adaptive and innate immune systems. Neutrophils, macrophages and natural killer cells (NKs) need Se to function effectively [17]. Se also plays a role as a NF-κB inhibitor, with consequent immune-modulation and anti-inflammatory action [18]. Se deficiency affects the activation and functions of T and B-lymphocytes, as T lymphocytes may be unable to proliferate in response to mitogens, and B lymphocytes may be ineffective to produce IgM and IgG [19]. An appropriate intake of Se might help alleviate oxidative stress and inflammation, and also reduce bacterial and viral infections [17].

The immune system is composed of two parts: the innate (epithelial barriers, lysozyme, interferon, complement, toll-like receptors, NK cells and phagocytes) and the adaptive (T and B lymphocytes and immunoglobulin) responses, which are integrated and cooperate with each other [20].

B-cells are responsible for the humoral immunity, also known as antibody-mediated immunity, through the differentiation into plasma cells that play a role in producing immunoglobulins. B lymphocytes undergo genetic modifications of their immunoglobulin genes to produce highly specific antibodies and five different immunoglobulin isotypes (IgG, IgM, IgA, IgE and IgD) [21]. Two thirds of all immunoglobulin produced by the body is IgA, which has an important influence in both humoral and mucosal immunity [22]. IgA exists in two forms: the monomeric form is free and dominant in the serum, while the dimeric form is wrapped by the secretory component and plays a fundamental role in mucosal immunity. Secretory IgA is more resistant to the proteolytic activity of bacteria, has an anti-inflammatory effect and protects against infectious agents and allergen sensitization, serving as an interface between the body and the microbiome [23].

Primary immunodeficiencies, also known as inborn errors of immunity, are a large and heterogeneous group of genetic diseases that impair the immune system. More than 400 diseases have been described [24]. In general, patients with primary immunodeficiencies are at risk for recurrent, prolonged, and sometimes life-threatening infections caused by several kind of pathogens, including opportunistic agents, autoimmunity, failure to thrive and malignancies [25,26]. An international expert committee composed of pediatric and adult clinical immunologists under the auspices of the International Union of Immunological Societies has provided the clinical and genetic classification of the inborn errors of immunity since 1970. Thus, these diseases are currently categorized into 10 groups, as follows: combined immunodeficiencies; combined immunodeficiencies with syndromic features; predominantly antibody deficiencies; diseases of immune dysregulation; congenital defects of phagocytes; defects in intrinsic and innate immunity; autoinflammatory diseases; complement deficiencies; bone marrow failure; and phenocopies of inborn errors of immunity [24].

Selective IgA deficiency (IgAD) is part of the predominantly antibody deficiencies group. IgAD is the most prevalent inborn error of immunity, with prevalence from about 1:3000 to even 1:150, depending on the population. According to the European Society for Immunodeficiency (ESID), the definition of primary IgAD was established as serum IgA levels less than 7 mg/dL, normal IgG and IgM and the exclusion of other causes of hypogammaglobulinemia in individuals aged over 4 years [27]. Many patients are oligosymptomatic, but less frequently they can present increased susceptibility for recurrent infections, and predisposition for allergies, gastrointestinal, endocrine and autoimmune disorders [22]. IgAD has been associated with lactase deficiency, celiac and Crohn’s disease, type 1 diabetes mellitus and rheumatoid arthritis. Allergies are present in 56% of individuals with IgAD, and asthma in 29.8%. Between 25.5 and 31.7% of individuals with IgAD develop systemic lupus erythematosus [22]. An increased risk for infections has been widely reported in individuals with IgAD. The most common infections are upper respiratory tract infections (40–90%), mainly viral, while bacterial infections are less frequent. Gastrointestinal tract infections are also common and include intermittent or chronic diarrhea due to Giardia lamblia [23]. There is no specific treatment for the disease, and management is directed to the clinical manifestations that may arise.

Our hypothesis is that patients with IgAD may have Zn and Se deficiency. Considering the importance of Zn and Se for several functions of the immune system, and the scarcity of previous studies that evaluated this association, the present study aimed to evaluate the nutritional status and the plasma and erythrocyte levels of these micronutrients in patients with IgAD. This assessment may be important for the implementation of changes in dietary behaviors and the possible supplementation of these micronutrients in order to modulate the immune system response and to reduce the susceptibility to infections.

## 2. Methods

### 2.1. Study Design

A cross-sectional, retrospective and descriptive study was conducted to evaluate some nutritional aspects of IgAD patients at the Clinics Hospital of Ribeirão Preto Medical School, University of São Paulo, Brazil.

### 2.2. Participants

Patients aged more than 4 years old with a confirmed diagnosis of selective IgAD, according to the ESID, were included. Age range was defined according to the recommendations of the World Health Organization, whereby children are individuals aged up to 10 years and adolescents are individuals between 10 and 20 years old [28]. Patients were selected among those who attended the Pediatric Allergy Outpatient Clinics at our hospital.

Individuals less than 4 years old, pregnant women, transitory or secondary IgAD, other secondary or primary immunodeficiencies, and the use of immunosuppressants or Zn and Se supplements were excluded.

This study was approved by the Ethics Committee of Clinics Hospital and patients were included after signing the consent and assent term to participate in the research (Protocol HCRP no 14444/2010).

### 2.3. Dietary Intake

All participants fulfilled a three days form with a detailed description of the ingested quantity and type of foods (food record). Forms were delivered to the participants or their parents. The form was returned during the next routine visit at the Outpatient Clinic. “Programa de apoio a Nutrição da Universidade Paulista de São Paulo “Nut Win” and “Tabela Brasileira de Composição de Alimentos da Universidade de Campinas (TACO-UNICAMP)” were used to estimate the daily intake of Zn and Se, according to the recommendations of the Reference Daily Intake (DRI).

### 2.4. Anthropometric Evaluation

Weight, height, measurement of triceps, biceps and subscapular skinfold thickness and mid upper arm circumference were performed in all patients. The nutritional status was based according to the classification of body mass index for age (BMI/age) for children and adolescents [29] and to BMI for adults [30]. The measurements of triceps skinfold thickness (TSF) and midarm muscle circumference (MAMC) were standardized according to Lohman [31] and classified according to Frisancho [32].

### 2.5. Laboratory Evaluation

Immunoglobulin measures were determined by automated nephelometry and repeated three times in different periods to confirm the values.

Zn and Se were measured in plasma and erythrocytes. Whole blood was collected in the morning in metal free tubes after 12 h of fasting.

The micronutrients concentration in plasma and erythrocytes was determined using a plasma couple mass spectrometer equipped with a reaction cell (DRC-ICP-MS ELAN DRCII, Perkin Elmer, Sciex, Norwalk, CT, USA) operating with high purity (99.999%) argon (Praxaair, Brazil). Each sample was diluted in 15 mL Falcon^®^ polypropylene tubes (Becton Dickison) at a 1:50 proportion with a solution containing 0.01%Triton X-100 (*v*/*v*), 0.5% HNO3 (*v*/*v*) and 10 µg/L^−1^ Rh of the internal standard Rh. The analytical calibration standards were prepared at a concentration ranging from 0 to 50 µg/L in the same diluent.

The quality control of the analyses was insured by the analysis of reference materials of the National Public Health Institute of Quebec, Canada. The analyses were carried out in the Laboratory of Toxicology and Essentiality of Metals, Faculty of Pharmaceutical Sciences of Ribeirão Preto, University of São Paulo.

The laboratory tests were carried out in the Central Laboratory of HCFMRP-USP according to standardized methods routinely used in the Institution. Total proteins and fractions were analyzed with the Konelab instrument (Wiener-lab^®^) and blood count and white cell count were performed using the ABX Pentra DX 120 instrument (HORIBA)^®^.

### 2.6. Statistical Analysis

The Shapiro Wilk test was used to assess data normality in the case of continuous variables, and the Kolmogorov Smirnoff test was used to determine the presence of possible associations between the variables of interest. When the normality was not rejected, parametric tests were used (Pearson correlation coefficient), and when there was no normality, nonparametric tests were used (Spearman correlation coefficient) [33].

*p*-value of less than 0.05 was considered significant. All the statistical analyses were performed with the SPSS (version. 22.0) and R (version. R-3.5.1) software.

## 3. Results

The study included 16 patients (9M:7F), 4 adults, 8 adolescents and 4 children with a confirmed diagnosis of IgAD. All patients presented at least one infection in the last 12 months and sinusitis was the most common infection. Table 1 shows the baseline demographic characteristics of the 16 patients.

Table 2 shows daily intake of nutrients in means and standard deviation (SD). All patients had an adequate intake of macronutrients according to the DRIs.

As observed in Table 2, 1 child, 2 adolescents and 1 adult had an inadequate intake of Zn, and only 1 adolescent had inadequate intake of Se.

Regarding the nutritional status, 75% of the children had an adequate BMI. Overweight and obesity were observed in 25% and 12,5% of the adolescents, respectively. All adults were classified as overweight or obese (Table 3).

Midarm muscle circumference (MAMC) and TSF were adequate for the majority of the patients. However, adults and adolescents would be classified as obese if only TSF was considered in the evaluation (152% and 189%, respectively).

Zn levels were evaluated for all patients in plasma and erythrocytes, as shown in Table 4.

We found normal levels of plasma Zn for the entire sample. However, all children, 75% of adolescents and 25% of adults had low levels of erythrocyte Zn.

**Figure 1:**
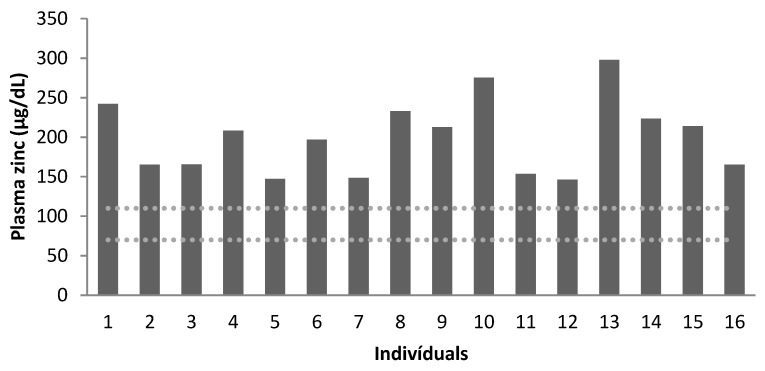
Plasma zinc concentration (µg/dL) of the patients (dotted lines correspond to the reference values, according to Mafra et al. [34]). Age group: patients 1 to 4: 7–9 years old; patients 5 to 12: 10–17 years old; patients 13 to 16: 19–25 years old.

**Figure 2:**
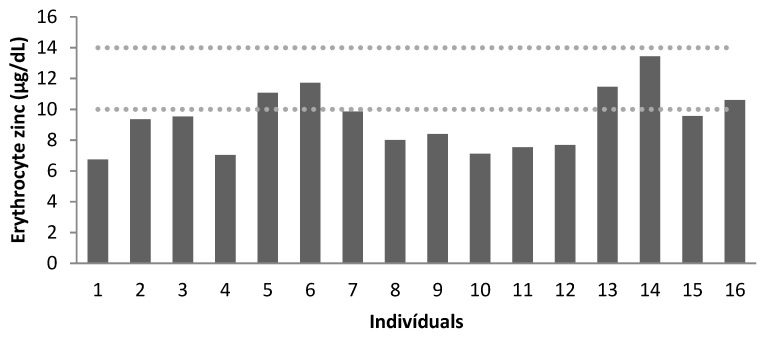
Erythrocyte zinc concentration (µg/dL) of the patients (dotted lines correspond to the reference values, according to Mafra et al. [34]). Age group: patients 1 to 4: 7–9 years old; patients 5 to 12: 10–17 years old; patients 13 to 16: 19–25 years old.

Figure 1 and Figure 2 demonstrate the concentration of plasma (μg/dL) and erythrocyte Zn (μg/dL), respectively. There was a moderate correlation between plasma and erythrocyte Zn levels (r2 = 0.547, *p* = 0.028).

No correlation was found between erythrocyte or plasma zinc and BMI, nutritional status or Zn intake.

Se levels were evaluated for all patients in plasma and erythrocytes. Plasma levels were higher than erythrocyte levels. All patients had low erythrocyte levels, as shown in Table 5.

**Figure 3:**
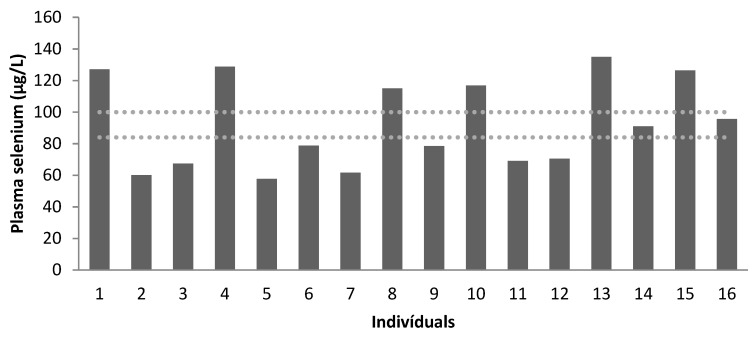
Plasma selenium concentration (µg/L) of the patients (dotted lines correspond to the reference values, according to Millán Adame et al. [35]). Age group: patients 1 to 4: 7–9 years old; patients 5 to 12: 10–17 years old; patients 13 to 16: 19–25 years old.

**Figure 4:**
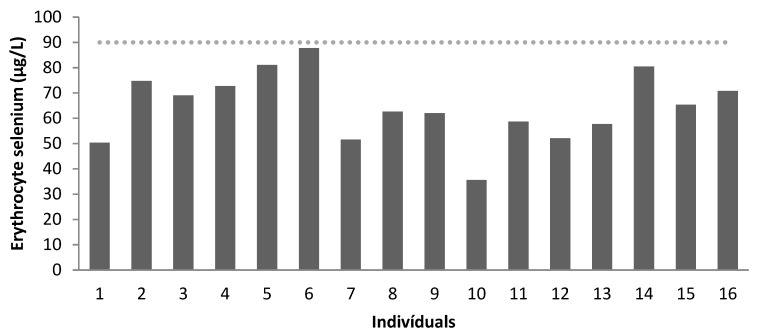
Erythrocyte selenium concentration (µg/L) of the patients (levels above the dotted line correspond to the reference value, according to Millán Adame et al. [35]). Age group: patients 1 to 4: 7–9 years old; patients 5 to 12: 10–17 years old; patients 13 to 16: 19–25 years old.

Figure 3 and Figure 4 show the concentration of plasma (μg/L) and erythrocyte Se (μg/L), respectively.

There was no correlation between Se levels and BMI, nutritional status or Se intake.

As for other relevant laboratory findings, all patients had normal levels of albumin and only 1 patient presented a mild reduction of the lymphocyte count.

Blood count, white cell count, albumin and total protein data are listed in Table 6. Albumin levels were within reference values for all patients. Another relevant biochemical indicator was total leukocyte count, since it evaluates immunological competence. Only one patient (6%) presented mild lymphocyte depletion, whereas all other subjects were within normal limits.

## 4. Discussion

Proper nutrition and adequate Zn and Se levels can help to maintain optimal immune function, reducing the impact of infections and other comorbidities. Accessibility to a proper amount of quality food is essential to maintain adequate body composition and immune function, especially for patients with inborn errors of immunity. The objective measurement of “enough food or nutrients” can be done by measuring the status of specific nutrients in the body, and is expressed in terms of their adequacy or deficiency [36].

In this cross-sectional and retrospective study, we assessed some biochemical parameters, the nutritional profile and the status of plasma and erythrocyte Zn and Se in patients with IgAD. We found a high proportion of patients that presented very low levels of Zn and Se in the erythrocytes. To our knowledge, this is the first study that investigated the plasma and erythrocyte status of Zn and Se in IgAD patients. Therefore, we highlight the relevance of the present study for the nutritional health of these patients.

Dos Santos-Valente et al. [37] evaluated 17 patients with common variable immunodeficiency, a severe inborn error of immunity, and 17.65% of the patients were considered malnourished and the serum and erythrocyte Zn levels were below normal. Kouhkan et al. [9] found different results. Approximately 3% of patients with different types of immunodeficiency, including IgAD, presented obesity, and 21% of patients presented with nutritional deficiency, according to the BMI. Also in this study, 86.8% of patients showed adequate levels of Zn in serum. On the other hand, Mariz et al. [38] evaluated adults with HIV infection and found a higher occurrence of overweight and obesity, similar to our study.

A high proportion of our patients had respiratory and intestinal infections in the last 12 months. Respiratory infections are an important cause of morbidity and mortality worldwide, and the importance of public health practices to reduce their spread is well established [39]. Zn has anti-infectious properties and a relevant role in defense against respiratory infections and regulating of the immune response in the respiratory tract [40].

Analyzing food consumption, Zn intake was considered adequate for the majority of patients, as only 4 individuals had intake inferior to the recommendation of DRI. However, Zn levels in the erythrocytes were deficient in 100% of children, 75% of adolescents and 25% of adults. Adequate Zn intake and homeostasis are essential for a healthy life, as Zn deficiency is associated with several immune disorders, metabolic and chronic diseases, as well as recurrent respiratory infections, malaria, HIV and tuberculosis [41]. On the other hand, the data intake was obtained by patient’s reports. Thus, these data may be underestimated or overestimated, depending on the quality of the reported information. As there is no storage compartment for these micronutrients in the human body, the micronutrients should be ingested daily in sufficient amounts [41].

The difference found between plasma and erythrocyte Zn concentration might be explained by changes in the erythrocyte levels according to several chronic conditions. Despite being the most used Zn biomarker, plasma level has low specificity and sensitivity as it can be influenced by recent changes in diet [41]. Erythrocyte Zn does not reflect these changes and can be considered a long-term biomarker [34]. This can justify why all patients evaluated in our study had both adequate intake and plasma levels, although erythrocytes levels were reduced. Zn supplementation may be more effective in children with deficient levels as compared to children with normal levels [42].

With respect to Se, almost all patients had an adequate intake. Se is acquired through food sources like nuts, breads, cereals, meat, fish, milk and dairy products [43]. Although some of these foods were adequately consumed by our sample, the data intake was obtained by patient’s reports, as for Zn.

Regarding the plasma and erythrocyte Se levels, our results were even more striking, as 100% of the patients had low erythrocyte levels, while 50% of children and 75% of adolescents had inadequate plasma levels. However, as for Zn, the plasma levels of Se can be considered an appropriate indicator of nutritional status in the short term, while erythrocytes levels may indicate a long-term nutritional status [35,44] Therefore, lower erythrocyte Se levels found in our patients might reflect a chronic deficiency state. Al Fify et al. [45] investigated patients with systemic inflammatory conditions and pointed out that erythrocytes measurements of certain micronutrients may be more reliable than plasma measurements.

Kouhkan et al. [9] had also demonstrated that Se deficiency was present in 37.5% of children with primary antibody immunodeficiency. When Se and Zn were evaluated in vegetarian adults, plasma levels were also higher compared to erythrocyte levels [46].

Further studies are necessary to evaluate if the normal range of Zn and Se levels in IgAD patients can be considered the same as in immunocompetent patients. This step would be important to assess if appropriate supplementation could reduce the recurrent infections.

Al Fify et al. [45] defined new standards to prevent the misdiagnosis and inadequate treatment of micronutrient deficiencies. They demonstrated that erythrocyte measurements can overcome the limitations of plasma measurements in patients with chronic inflammatory diseases.

Zn has an important role in the formation, maturation and function of T and B cells. Moreover, Zn deficiency can cause reduced maturation of these cells, resulting in reduced antibody production. Adequate Se levels are also fundamental for immune system function, since selenoproteins can regulate inflammation and immunity [15]. Patients with inborn errors of immunity, including IgAD, are at risk for infections and autoimmunity. Therefore, it is essential to evaluate their nutritional status for seeking possible mineral deficiencies that may be supplemented in order to guarantee a more effective functioning of the immune system.

The main strength of this study is that Zn and Se were measured in erythrocytes for all patients who were evaluated by the same investigator throughout the study.

We can point to some limitations of this study. The low number of patients, despite IgAD being considered an inborn error of immunity and therefore being included in the rare diseases group. This was a retrospective study. The lack of a control group.

Micronutrient deficiencies are frequently found in clinical practice, in both children and adults. However, these deficiencies are often underrecognized. Therefore, clinicians should be aware of the risk factors and act properly [44]. Further studies should explore the impact of specific micronutrient supplementation for patients with IgAD and other inborn errors of immunity.

## 5. Conclusions

The erythrocyte levels of Zn and Se were low in IgAD patients, as compared with the reference values by age range. Our findings suggest the need for monitoring both the intake and erythrocyte levels of these micronutrients in this group of patients.

## Figures and Tables

**Table 1:** Demographic characteristics of the patients.

Variables	*n* (%)
**Sex**	
Male	9 (56)
Female	7 (44)
**Age Distribution**	
Children	4 (25)
Adolescent	8 (50)
Adult	4 (25)
**Outpatient infections in the last 12 months**	
Acute otitis media	3 (19)
Sinusitis	8 (50)
Diarrhea	5 (31)
Pneumonia	2 (12)
Other	7 (44)

**Table 2:** Daily intake of nutrients of the patients.

	4–10 Years Mean ± SD	11–19 Years Mean ± SD	20–25 Years Mean ± SD
Energy (kcal/day)	1533.3 ± 338.6	1852.5 ± 865.1	2304.1 ± 379.9
DRI (kcal/day)	1759.5 ± 171.5	2272.6 ± 588.5	3193.87 ± 634.3
Protein (g/day)	65.4 ± 20.5	74.8 ± 21.9	100.11 ± 22.6
DRI (g/day)	20.7 ± 4.6	37.9 ± 11.4	58.2 ± 8.3
Protein (%)	16.8 ± 1.9	16.6 ± 4.7	16.6 ± 1.9
DRI (%)	10–35	10–35	10–35
Carbohydrates (%)	57.2 ± 4.9	57.6 ± 5.9	61.3 ± 7.2
DRI (%)	45–65	45–65	45–65
Lipids (%)	26.9 ± 3.1	26.0 ± 1.2	22.5 ± 5.6
DRI (%)	25–35	25–35	20–35
Zn (mg/day)	8.2 ± 2.7	9.8 ± 3.2	11.3 ± 2.6
DRI (mg/day)	4–7 (EAR)	7–8.5 (EAR)	6.8–9.4 (EAR)
Se (µg/day)	64.5 ± 26.1	69.7 ± 21.4	96.7 ± 14.4
DRI (µg/day)	23–35 (EAR)	35–45 (EAR)	45 (EAR)

DRI: Dietary Reference Intake.

**Table 3:** Nutritional status of the patients.

	Groups		
Nutritional Status	Children * n (%)	Adolescents * n (%)	Adults ** n (%)
Low weight	1 (25)	-	-
Eutrophy	3 (75)	5 (62)	-
Overweight	-	2 (25)	2 (50)
Obesity	-	1 (12)	2 (50)

* Eutrophic: BMI/age between 3rd and 85th percentile. Low weight: BMI/age below 3rd percentile. Overweight: BMI/age between 85th and 97th percentile. Obesity: BMI/age above 97th percentile (WHO [29]). ** Eutrophic: BMI between 18.5 and 25 kg/m^2^. Low weight: BMI bellow 18.5 kg/m^2^. Overweight: BMI between 25 and 30 kg/m^2^. Obesity: BMI above 30 kg/m^2^ (WHO [30]).

**Table 4:** Zinc concentration in plasma (µg/dL) and erythrocytes (µg/dL) of the patients.

Zn	Children		Adolescents		Adults	
	Plasma	Erythrocytes	Plasma	Erythrocytes	Plasma	Erythrocytes
Recommendation *	70–110	10–14	70–110	10–14	70–110	10–14
Mean	195	8	189	9	225	11
Below Normal	0	100%	0	75%	0	25%
Normal	100%	0	100%	25%	100%	75%

* Based on Mafra et al. [34].

**Table 5:** Selenium concentration in plasma (µg/L) and erythrocytes (µg/L) of the patients.

Se	Children		Adolescents		Adults	
	Plasma	Erythrocytes	Plasma	Erythrocytes	Plasma	Erythrocytes
Recommendation *	84–100	>90	84–100	>90	84–100	>90
Mean	95.86	66.70	81.03	61.43	111.96	68.57
Below Normal	50%	100%	75%	100%	0	100%
Normal	50%	0	25%	0%	100%	0%

* Based on Millán Adame et al. [35].

**Table 6:** Laboratory parameters of the patients with IgAD.

	Children		Adolescents		Adults	
	Mean	SD	Mean	SD	Mean	SD
Hemoglobin (g/dL)	13.28	0.83	12.94	0.52	16.23	2.09
Hematocrit (%)	40.50	2.65	38.88	1.36	48.75	6.50
MCV (µ/mm^3^)	79.75	10.53	87.75	6.09	90.75	5.68
MCH (pg)	26.28	3.59	29.15	1.93	30.40	1.79
Leukocytes (cells × 10^3^/mm^3^)	8.42	4.36	7.72	2.62	7.82	0.95
Total lymphocytes (cells × 10^3^/mm^3^)	2.35	0.97	2.54	0.78	2.14	0.8.5
Total proteins (g/dL)	7.35	0.29	7.55	0.41	7.16	0.73
Albumin (g/dL)	4.27	0.44	4.21	0.40	4.13	0.51

SD: standard deviation; MCV: Mean corpuscular volume; MCH: Mean corpuscular hemoglobin.

## Data Availability

The data presented in this study are available on request from the corresponding author.
